# Absolute pitch can be learned by some adults

**DOI:** 10.1371/journal.pone.0223047

**Published:** 2019-09-24

**Authors:** Stephen C. Van Hedger, Shannon L. M. Heald, Howard C. Nusbaum

**Affiliations:** 1 Department of Psychology, The University of Chicago: Chicago, IL, United States of America; 2 Center for Practical Wisdom, The University of Chicago: Chicago, IL, United States of America; University of Haifa Faculty of Education, ISRAEL

## Abstract

Absolute pitch (AP), the rare ability to name any musical note without the aid of a reference note, is thought to depend on an early critical period of development. Although recent research has shown that adults can improve AP performance in a single training session, the best learners still did not achieve note classification levels comparable to performance of a typical, “genuine” AP possessor. Here, we demonstrate that these “genuine” levels of AP performance can be achieved within eight weeks of training for at least some adults, with the best learner passing all measures of AP ability after training and retaining this knowledge for at least four months after training. Alternative explanations of these positive results, such as improving accuracy through adopting a slower, relative pitch strategy, are not supported based on joint analyses of response time and accuracy. The results also did not appear to be driven by extreme familiarity with a single instrument or octave range, as the post-training AP assessments used eight different timbres and spanned over seven octaves. Yet, it is also important to note that a majority of the participants only exhibited modest improvements in performance, suggesting that adult AP learning is difficult and that near-perfect levels of AP may only be achievable by subset of adults. Overall, these results demonstrate that explicit perceptual training in some adults can lead to AP performance that is behaviorally indistinguishable from AP that manifests within a critical period of development. Implications for theories of AP acquisition are discussed.

## Introduction

Absolute pitch (AP), also called “perfect pitch”, is the rare ability to name any musical note without the aid of a reference note [1–3]. The question of how individuals acquire this ability continues to be a matter of debate. The most widely accepted theory is that “genuine” AP ability can only be developed as a result of an early critical period of learning (the *critical period* theory) [4,5]. However, this *critical period* theory of AP is bolstered in large part by the lack of conclusive evidence that AP can be learned by any post-critical-period adults, thus resting on null findings [6–9]. Critical periods in AP development have also been supported in principle through training studies. For example, one study found that children outperformed adults in a learning paradigm that focused on learning a single note [10]. More recently, a training study provided proof-of-concept demonstration of critical periods in AP by demonstrating that critical period for learning AP could be “re-opened” for adults via a pharmacological intervention [11]. Yet, it is important to note that the learning observed in these critical period training studies was well below thresholds typically used to identify the level of performance that is characteristic of AP.

Here, we directly test the hypothesis of a critical period for AP acquisition through intensive AP training in a post-critical-period-adult sample. One key difference between this study and previous AP training studies [7,12–14] is that participants were selected specifically based on auditory working memory (WM) ability. Recent research shows that individual differences in auditory WM predict how well adults can learn AP categories from a single brief training session, although after approximately 45 minutes of training this level of performance was not at the level of typical genuine AP ability, even for the highest-performing individuals [15]. Given this empirical evidence, we tested whether providing substantially more AP training for adults with high auditory WM abilities can produce performance comparable to a genuine AP listener (i.e., virtually perfect and fast note identification), including retention of learning (over a timescale of months) and accurate performance across a range of instrumental timbres and octaves. This type of evidence, even in a single adult without prior AP ability, would inform our understanding of the *critical period* theory of AP.

As an alternative to the *critical period* theory, AP can be conceptualized as an auditory skill [16] that is shaped by both short- and long-term experiences [16,17]. This view (which we refer to as the *skill acquisition* theory) predicts that listeners should be able to improve AP performance through explicit perceptual training at any age, with at least some individuals exceeding typical thresholds for genuine AP inclusion post-training. If, however, individuals are only able to modestly improve in AP categorization, with no individual reaching genuine AP performance post-training, this suggests that there might be fundamental limits that constrain performance as would be expected under a critical period framework. Thus, the question of whether a post-critical-period adult can learn AP has important implications for understanding individual differences in AP performance, the underlying mechanisms of AP, and the importance of environmental factors on developing and maintaining AP ability.

Six adult participants completed an eight-week AP training regime. Prior to training, participants completed tests of auditory WM performance, short-term memory (STM), and AP ability. The eight-week AP training program was divided into two phases lasting four weeks each. In both phases of training, participants had to complete three training protocols four times each week, which amounted to approximately 4 hours of training per week (32 hours in total). Every week, participants also completed an AP test in which isolated notes were categorized without feedback. In the second phase of training, we added an additional test that required participants to label the key signature of a presented melody without feedback. At the end of the eight-week training program, participants were given the same AP tests that were administered prior to training, and in addition given an AP test not previously administered but widely used in prior research. Approximately four months later, we retested participants on the same AP tests as those given post-training to assess whether learning remained stable.

Given the theoretical importance of even a single participant reaching performance levels comparable to a genuine AP possessor post-training, the present experiment does not adopt a typical inferential statistical approach (i.e., performed at the group level). This is not to say that such an approach is not important in the context of adult AP training; it is simply not the focus of the current experiment. Rather, the present experiment borrows from case study [18] and “small *n*” [19] designs, treating the individual as the unit of analysis. This approach is particularly apt given the extensive training and testing done at the individual level and our goal to assess whether even a single adult can acquire AP outside of a critical period. While this approach limits our ability to comment on which post-critical-period adults may have an easier time acquiring genuine AP-level performance, the current approach stands as a robust test for assessing whether post-critical-period AP acquisition is even possible.

## Method

### 2.1 Participants

Six participants (three female, three male) participated in the experiment (*M* = 23.33, *SD* = 2.94 years old, age range: 18–26). The participants consisted of a convenience sample (i.e., all participants had primary or secondary affiliations with the research lab of the senior author). Superior auditory memory abilities were initially provided via self-report, although all participants’ auditory short-term and working memory abilities were tested and verified to be superior prior to training (see Section 3.1.1). All participants reported at least some musical training (see Table 1), as do many people with and without AP, although none of the participants self-reported possessing AP nor would qualify as possessing AP under any extant performance standards.

**Table 1:** Summary of participants’ musical training backgrounds.

Participant	Age of Music Onset (years)	Primary Instrument	Training on Primary Instrument (years)	Actively Playing	Current Playing (hrs. / week)
S1	3	Violin	11	Yes	> 6
S2	7	Piano	8	No	< 1
S3	6	Piano	15	Yes	3–4
S4	7	Piano	15	No	< 1
S5	6	Violin	20	Yes	3–4
S6	8	Piano	17	Yes	> 6

Note: Age of music onset is the reported age at which participants formally began musical training.

### 2.2 Ethical approval and informed consent

The research was conducted in accordance with the protocol approved by the Social and Behavioral Sciences Institutional Review Board at the University of Chicago. All participants provided written consent and were compensated for their participation in the experiment.

### 2.3 Task description and materials

#### 2.3.1 AP training program

The absolute pitch training program was eight weeks in duration. There were two phases to the experiment (each lasting four weeks). Both phases consisted of three training programs that participants had to complete four days every week. Participants were tested every Friday, meaning that participants could complete their weekly training programs Saturday through Thursday.

**2.3.1.1 First phase:** Two of the First Phase training programs were meant to emphasize speed in classifying absolute pitches, while the third was meant to emphasize accuracy. In the first program, nicknamed “Simple Speed,” participants completed 14 trials in which they had to rapidly identify a target note. At the beginning of each trial, participants saw a note name presented in the center of the screen for 1500 ms (e.g., C). This was the target note for the trial. During the presentation of the note name, participants also heard a sung version of the target note, which was enunciated with the category label. After this 1500 ms setup for the trial, they heard a string of 16 notes. All notes were 1000 ms in duration, and were taken from a C-major scale (i.e., white keys only) spanning a one-octave range (C [4] to B [4]). Non-target notes were also taken from a one-octave range and consisted of only white keys. The notes were synthesized with a piano timbre. Within these 16 notes, 25% (4 of 16) were the target note, while 75% (12 of 16) were non-target notes. Target notes were randomly interspersed with non-target notes. Participants had to press the spacebar as quickly as possible whenever they heard the target note established at the beginning of the trial. From the onset of the note, participants had 1750 ms to respond, and participants could make their response while the note was still playing. As soon as participants pressed the spacebar, they were redirected to the feedback screen (containing generic feedback of either “That was the target note,” printed in green, or “That was not the target note”, printed in red), which remained on the screen for 500 ms. If participants did not press the spacebar, they were redirected to the feedback screen at the end of the response window of 1750 ms. As such, the interval between successive notes was dependent on how quickly participants made their response, with the maximum interval between successive notes in a trial being 2250 ms (up to 1750 ms to respond to the note and 500 ms of feedback). There was a 1500 ms rest period between trials. Each of the seven target notes (C, D, E, F, G, A, and B) were presented in this ascending sequential order, twice.

The second program, nicknamed “Complex Speed,” followed the same general procedure as “Simple Speed” with the following differences. First, the presentation of the 16 notes was faster, meaning that participants had to respond more quickly. Despite all notes having the same duration as those used in “Simple Speed” (1000 ms), participants were restricted to just 1250 ms following the note onset to make their response. While the interval between successive notes was still dependent on how quickly participants made their response, the reduction of the response window shortened the maximum interval between successive notes in a trial to 1750 ms (up to 1250 ms to respond to the note and 500 ms of feedback). Second, the notes (both target and distractor) could come from a piano, flute, or guitar timbre. Third, the octave range of both the target and distractor notes was expanded relative to the SS task (C [3] to B [5]), though both targets and distractors were still only sampled from white-key notes. Timbre and octave were randomized within a trial (i.e., participants were not able to predict the timbre or octave of the next note given the current note). Target notes were still presented in an ascending sequential order (C, D, E, F, G, A, B), twice, for 14 total trials during each session.

The third program, nicknamed “Accuracy Training,” emphasized correct note category identification over speed of identification. There were two blocks, consisting of 48 trials each (96 trials total). The general procedure was the same for both blocks. On each trial, participants would hear an isolated note, which was presented simultaneously with a response screen. The response screen displayed all twelve note-category options, arranged in a pitch wheel. Participants then had an unlimited amount of time to click on the corresponding note name with the mouse. After each response, participants would receive feedback, in which the correct note name was highlighted, and participants reheard the note. The first block consisted of piano notes, spanning a two-octave range (C [4] to B [5]). Unlike the “Simple Speed” and “Complex Speed” tasks, all 12 categories were explicitly trained. Since there were 24 total piano notes (12 note categories x 2 octaves), each note was presented twice in a random order. The 48 notes in the second block were randomly selected from 96 possible notes (24 piano, 24 cello, 24 clarinet, and 24 harpsichord). Participants heard 1000 ms of noise played between trials.

Every Friday, participants would have to complete a weekly test (WT) of AP ability. The WT was similar to “Accuracy Training” with two exceptions. First, participants did not receive feedback. Second, the second block was expanded to 120 possible notes (24 piano, 24 cello, 24 clarinet, 24 harpsichord, and 24 square wave).

**2.3.1.2 Second phase:** We replaced the speeded tasks (“Simple Speed” and “Complex Speed”) after Week 4 with a more difficult speeded task, nicknamed “Hypercomplex Speed.” This new task followed the same general procedure as the speeded tasks from the First Phase, with the following exceptions. First, on each trial, participants heard a string of 32 notes, with a more restrictive response window equal to the length of the audio files (1000 ms). While the interval between successive notes was still dependent on how quickly participants made their response, the maximum interval between successive notes in a trial was reduced to 1500 ms (up to 1000 ms to respond to the note and 500 ms of feedback). Within these 32 notes, 12.5% (4 of 32) were the target note, while 87.5% (28 of 32) were non-target notes. Second, the timbre range was expanded relative to the CS (with the addition of harpsichord, clarinet, cello, and square tones). Third, the distractor notes were sampled from all 12 categories (i.e., white-key notes and black-key notes). This expansion of distractor notes to all note categories removes the potential cue of tonal function, which was present in the First Phase speeded tasks (as all notes in the “Simple Speed” and “Complex Speed” tasks, target and distractor, could be interpreted in a C major / A natural minor tonal framework). Targets were still sampled from white-key notes, though they were randomized (not presented in an ascending sequential order, as was the case in the “Simple Speed” and “Complex Speed” tasks) and could come from any timbre. The octave range for all notes, both target and distractor, remained C [3] to B [5]. There were 14 total trials for each session.

We also introduced a novel task during the Second Phase nicknamed “Name That Key.” This task followed the same general procedure as the “Accuracy Training” task. Each session, participants heard 15 music recording excerpts, which were randomly selected from a database of 300 total recordings. Similar to the “Accuracy Training” task, participants had to decide on the key signature of the recording by clicking on a pitch wheel with the 12 pitch categories arranged around a circle. After each selection, participants would see a specific feedback screen, in which the correct note name was highlighted on the pitch wheel. Additionally, participants heard the tonic note of the key signature (e.g., C) played during feedback. Participants heard 1000 ms of noise between trials. Participants also completed the “Accuracy Training” task during the Second Phase, which was identical to the version presented in the First Phase.

Every Friday, participants would have to complete the same WT from the First Phase, in addition to a new test, nicknamed the “Name That Key Test” (NTKT). The NTKT required participants to judge the key signature of 12 folk songs. During the task, each pitch class was represented by a key signature one time (i.e., one folk song played in C, one folk song played in C#, etc.), and feedback was not provided. The mode of the key signature was preserved (e.g., “Mary Had a Little Lamb” could be represented in C *major*, C# *major*, etc.). In other words, the relative pitches were preserved. The randomized assignment of folk melody to key signature was hard coded into the script, as we did not want any given folk song to play in the same key signature across weeks.

The isolated musical note stimuli used in training were created using Reason Music Production Software (Propellerhead: Stockholm, Sweden). Musical notes were sampled from real instruments and were digitized at a 44.1 kHz sampling rate with 16-bit depth, were 1000 ms in duration, and were root mean square normalized to a level of -5 dB FS. We used 160 total notes (from seven instrumental timbres) throughout training. For the music recording stimuli used in the “Name That Key” task, we recorded 15-second excerpts from 300 popular pieces of music (e.g., pop songs, movie themes). For the NTKT, we recorded simple piano melodies of folk songs using Reason. We then transposed and exported each folk melody in every key. The explicit absolute pitch training and testing programs were run on participants’ personal computers using Open Sesame software [20]. All stimuli and training scripts are available on the Open Science Framework.

#### 2.3.2 Tests of absolute pitch

We assessed AP ability before and after the training program using several tests of AP. One of these tests (hereafter referred to as the UCSF Test) has been used in several prior studies of AP [21,22], and could be accessed through the University of California San Francisco AP website. We recorded the audio stimuli (40 piano tones, 40 sine wave tones) from the website and administered the test offline and in the lab to monitor the participants, minimizing the possibility of using non-absolute cues or strategies such as humming to artificially inflate their scores. The piano notes ranged from C [1] (32.70 Hz) to G# [7] (3322.44 Hz), whereas the sine notes ranged from C [2] (65.41 Hz) to G# [8] (6644.88 Hz). We removed the four highest tones for the sine wave test and the four lowest tones for the piano test, which is standard for scoring the UCSF Tests. Participants identified each note in writing.

Both the UCSF Piano and Sine Tests consisted of 40 trials. Each tone lasted 1000 ms with approximately a 2250 ms inter-stimulus interval. There was a longer break (10 s) after every 10 notes, which gave participants the opportunity to check to make sure that they were categorizing the correct trial number. Under no circumstances were participants allowed to repeat any of the notes. Participants were randomly assigned to take either the Piano or Sine Test first.

The second AP test (hereafter referred to as the UCSD Test) was taken from Diana Deutsch’s website (deutsch.ucsd.edu), and has been used to assess AP ability across a wide variety of participants [23]. To minimize the use of relative pitch as a cue, all intervals between successive notes were larger than an octave (i.e., at least 13 semitones apart). The UCSD Test consisted of 36 scored piano notes, which spanned from C [3] (below middle C) to B [5] (almost three octaves above middle C) divided into three blocks of 12 notes. The first four notes were not scored and were designated as practice trials, which is standard for this test. Each piano note lasted approximately 500 ms with an inter-stimulus interval of approximately 3750 ms. After each block of 12 notes, there was an extended break of approximately 18 s, which gave participants the opportunity to check to make sure that they were categorizing the correct trial number. Under no circumstances were participants allowed to repeat any of the notes. We downloaded the audio file from the website and administered the test offline and in the lab to monitor the participants, minimizing the possibility of using non-absolute cues or strategies such as humming to artificially inflate their scores. The participants identified each note in writing. Answer keys for both the UCSD and UCSF Tests were created by calculating the fundamental frequency of each note in Adobe Audition. These fundamental frequencies were then checked against a piano keyboard.

The AP test we developed for the purposes of this study (hereafter referred to as the Chicago Test) was identical to the WT from training. Participants completed a Piano Block (48 trials), followed by a Multiple Timbres Block (48 trials) on a computer in an untimed fashion. We treated the Piano Block separately from the Multiple Timbre Block. All response time analyses associated with the Chicago Test remove outlier trials (greater than three standard deviations above the mean response time). The outlier cutoffs were separately calculated for each session and for each participant.

#### 2.3.3 Tests of auditory working memory

The implicit note memory (INM) task has been previously used as a test of auditory working memory precision[15], and has also been associated with both explicit and implicit AP representations[15,24]. On each trial, participants heard a brief (200 ms) sine wave target note, which was then masked by 1000ms of noise. Participants then had to adjust a starting note, by clicking on upward and downward arrows on the computer screen, to try to recreate the originally heard target note. The arrows moved the pitch either 10 or 20 cents up or down, depending on whether participants were clicking on the smaller arrows (10 cents) or larger arrows (20 cents). When participants believed that they had successfully recreated the original target note, they pressed a key to move onto the next trial.

There were 27 sine waves in the distribution. The lowest frequency was 471.58 Hz, corresponding to a 20-cent sharp A# [4] and the highest frequency was 547.99 Hz, corresponding to a 20-cent flat C# [5]. The intermediary frequencies were evenly spaced in 10-cent increments. On each trial, participants had to recreate one of 10 possible targets (corresponding to the odd-numbered pitches in the distribution, spanning from pitch 5 to 23, inclusive). The frequencies of the targets were thus 482.60 Hz, 488.21 Hz, 493.88 Hz, 499.62 Hz, 505.42 Hz, 511.30 Hz, 517.24 Hz, 523.25 Hz (C [5]), 529.33 Hz, and 535.48 Hz. Participants recreated these targets by starting from one of four fixed locations, located outside of this pitch range. There were two starting locations below this target distribution (pitch 1: 471.58 Hz and pitch 3: 477.06 Hz) and two starting locations above this target distribution (pitch 25: 541.70 Hz and pitch 27: 547.99 Hz). In recreating the target note, participants were bounded by this distribution (i.e., participants could not adjust the pitch lower than 471.58 Hz or higher than 547.99 Hz, and could only move the note in 10- or 20-cent increments). Participants randomly heard all combinations of target / starting location twice, resulting in 80 trials (10 target notes x 4 starting notes x 2 repetitions). The INM task was run using the Psychophysics Toolbox in Matlab. [25,26]

The auditory n-back (NB) task required participant to monitor the identity of spoken letter strings and remember whether the current spoken letter matched the letter presented *n* trials previously. All participants completed a 2-back and a 3-back task. For all the trials in which the current letter did not match the spoken letter presented n trials previously, participants were instructed to press a button labeled “Not Target.” Both the 2-back and 3-back consisted of 90 trials (three runs of 30 spoken letters). Letters were spoken sequentially, with an inter-stimulus-interval of 3000 ms. Targets occurred one-third of the time, while non-targets occurred two-thirds of the time. Before the 2-back and 3-back, participants completed a practice round of 30 trials to familiarize themselves with the task. The NB was run in E-Prime 2.0 (Psychology Software Tools: Sharpsburg, PA).

We assessed auditory short-term memory (STM) using the auditory digit span (ADS) task. For the ADS task, participants initially heard three trials of five-number strings (e.g., 27483). There was 1000 ms of silence between spoken numbers. Participants correctly needed to identify a majority (at least two) of the five-number strings in order to advance to six-number strings. If participants could not correctly identify a majority of the five-number strings (attaining zero or one correct answer), they were given three trials of four-number strings. This process of adding or removing a number based on performance repeated eight times. Thus, a perfect performance would yield a digit span score of 13 (5+8), while a completely inaccurate performance would yield a digit span score of one. The ADS was run in E-Prime 2.0.

#### 2.3.4 Questionnaires

Participants filled out a music experience questionnaire at the completion of training, which asked about primary music experience, the number of instruments played (as well as the number of years of active musical instruction on each instrument), and the age of beginning musical instruction. Additionally, participants filled out a follow-up questionnaire, which specifically asked about participants’ explicit AP training in the time between posttest and follow-up.

### 2.4 Procedure

After providing informed written consent, participants completed the NB, INM, and ADS measures, which were followed by the AP Tests (UCSF and Chicago). These were completed in the lab over in a single session. Participants were then given portable drives containing the AP Training Program, which contained detailed instructions for completion. Participants were additionally walked through the general training protocol by the experimenter.

Over the next eight weeks, participants completed both phases of their training program as specified by the instructions. One participant (S5) was unable to complete three days of training in Week 7. Every Friday, participants completed their weekly tests. Participants uploaded their data to a secure server after each week.

Within one week of completing the eight-week training program, participants returned to the lab to complete the external AP tests (UCSF and UCSD), in addition to a musical experience questionnaire. We treated participants’ final WT as their Chicago posttest, as this was completed on the final day of the training program. The follow-up test, which was identical in design to the posttest, was conducted approximately four months after training had ended depending on availability of the participants. No feedback was given at any point during testing. Around the same time as the follow-up test, participants completed an online questionnaire that assessed their musical activities in the period between the posttest and follow-up, including explicit AP practice.

## Results

### 3.1 Pre-training

#### 3.1.1 WM and STM assessments

Results from the pretest session confirmed that all participants scored well on the measures of auditory WM and STM. The average auditory n-back (NB) scores (using d-prime measurements [27]) were 4.05 (*SE*: 0.17) for the 2-back, and 3.52 (*SE*: 0.32) for the 3-back. For reference, in a previous study [15] the average auditory 2-back score was 3.37 (*SE*: 0.16) and the average 3-back score of 2.27 (*SE*: 0.19) from a sample primarily consisting of University of Chicago undergraduates. The average error in the implicit note memory (INM) task was 2.75 (*SE*: 0.30) steps (corresponding to 27.5 cents) meaning that participants were able to recreate a briefly presented sine wave tone within approximately one-quarter of one semitone. For reference, the error of genuine AP possessors performing the identical task is on average 2.44 (*SE*: 0.19) steps [28]. In an auditory digit span (ADS) task, participants correctly recalled an average of 8.83 (*SE*: 0.65) spoken digits, which was between the range of non-AP musical controls (8.1) and genuine AP possessors (10.0) previously reported [29].

#### 3.1.2 AP assessments

**Fig 1:**
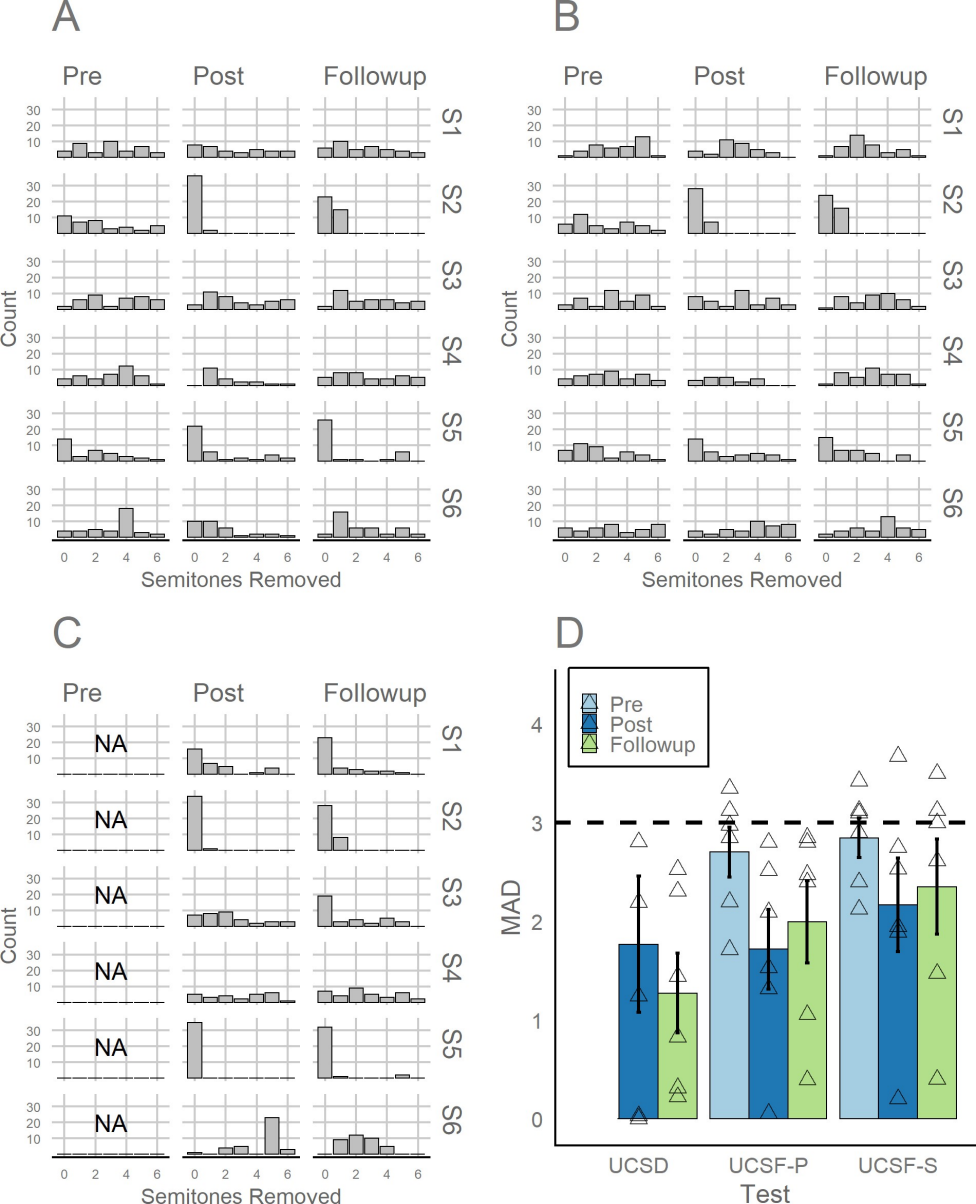
Distribution of semitone errors for each participant (S1-S6) and each testing session (Pre, Post, and Follow-Up) for the UCSF Piano Test (A), UCSF Sine Test (B), UCSD Test (C), and mean absolute deviation (MAD) across all participants for these tests (D). The dashed line in Panel D represents a MAD value that would be expected by random guessing (i.e., uniform distribution of errors ranging from 0 to 6 semitones removed from the correct note).

Prior to training, no participant reached or exceeded the AP cutoffs that have been previously established in the external UCSF AP Test [22]. We scored the UCSF Test by giving full credit for a correct answer and three-quarters credit for answers that fell within one semitone of the correct answer, as this scoring rubric has been previously adopted for this test [21]. For all reported AP tests, we also calculated participants’ mean absolute deviation (MAD) from the correct note in semitones, with scores of 0 reflecting perfect performance (i.e. 0 semitones removed from the correct note) and scores of 3 reflecting random guessing (i.e. uniform distribution of errors ranging from 0 to 6 semitones removed). Out of a maximum score of 36, prior to training, participants averaged 8.92 points in the Sine Test (MAD: 2.79, *SE*: 0.24) and 10.38 points in the Piano Test (MAD: 2.62, *SE*: 0.29). This level of performance was below the cutoff for the highest designation of AP (*AP-*1), defined as a minimum score of 24.5 on the Sine Test, as well as below the cutoff for the lowest AP designation (*AP-4*), defined as a minimum score of 27.5 on the Piano Test, as specified by the creators of the test [22]. Moreover, all participants scored within the range of non-AP participants from prior investigations using this test. The distribution of pre-training responses for each participant is shown in Fig 1A (Piano) and Fig 1B (Sine), with average MAD represented in Fig 1D. All participant scores (out of 36) are reported in Table 2.

**Table 2:** Performance measures across the Chicago Piano (C-P) and Multiple Timbre (C-MT) Blocks, the UCSF Piano (UCSF-P) and Sine (UCSF-S) Tests, as well as the UCSD Test.

*Pretest* Participant	C-P	C-MT	UCSF-P	UCSF-S	UCSD
S1	25.00% (7.14s)	25.00% (4.08s)	9.25	3	*NA*
S2	62.50% (2.81s)	52.08% (3.19s)	16.25	12.75	*NA*
S3	14.58% (4.13s)	12.50% (4.36s)	5.75	7.5	*NA*
S4	20.83% (7.71s)	12.50% (7.08s)	8.5	8.5	*NA*
S5	70.83% (3.31s)	68.75% (2.90s)	15.5	13.5	*NA*
S6	6.25% (6.90s)	4.17% (6.44s)	7	8.25	*NA*
*Posttest* Participant	C-P	C-MT	UCSF-P	UCSF-S	UCSD
S1	52.08% (7.08s)	54.17% (6.25s)	13.25	4.75	41.67%
S2	97.92% (1.53s)	100% (1.48s)	34.5	29.5	94.44%
S3	37.50% (2.43s)	31.25% (2.16s)	10.5	11.5	19.44%
S4	47.92% (4.48s)	52.08% (5.51s)	7.5	6	11.11%
S5	100% (2.81s)	100% (2.79s)	24.75	17.75	97.22%
S6	0% (2.32s)	0% (2.58s)	16.75	5.5	2.78%
*Follow-up* Participant	C-P	C-MT	UCSF-P	UCSF-S	UCSD
S1	56.25% (6.48s)	52.08% (5.84s)	13.5	6.25	63.89%
S2	93.75% (1.73s)	91.67% (2.02s)	30.75	32.75	77.78%
S3	33.33% (2.65s)	47.92% (2.34s)	9.5	7	52.78%
S4	18.75% (6.21s)	22.92% (6.34s)	11	7	19.44%
S5	93.75% (3.01s)	91.67% (2.81s)	24.75	18.25	91.67%
S6	2.08% (2.92s)	0% (3.07s)	13.25	5	0%

Note: Parentheses following the C-P and C-MT percentages represent mean response time. UCSF values represent performance (out of 36 scored trials), where full credit was given for correct answers and three-quarters credit was given for incorrect answers within one semitone of the correct answer. UCSD values represent percentage correct (no credit for semitone errors).

**Fig 2:**
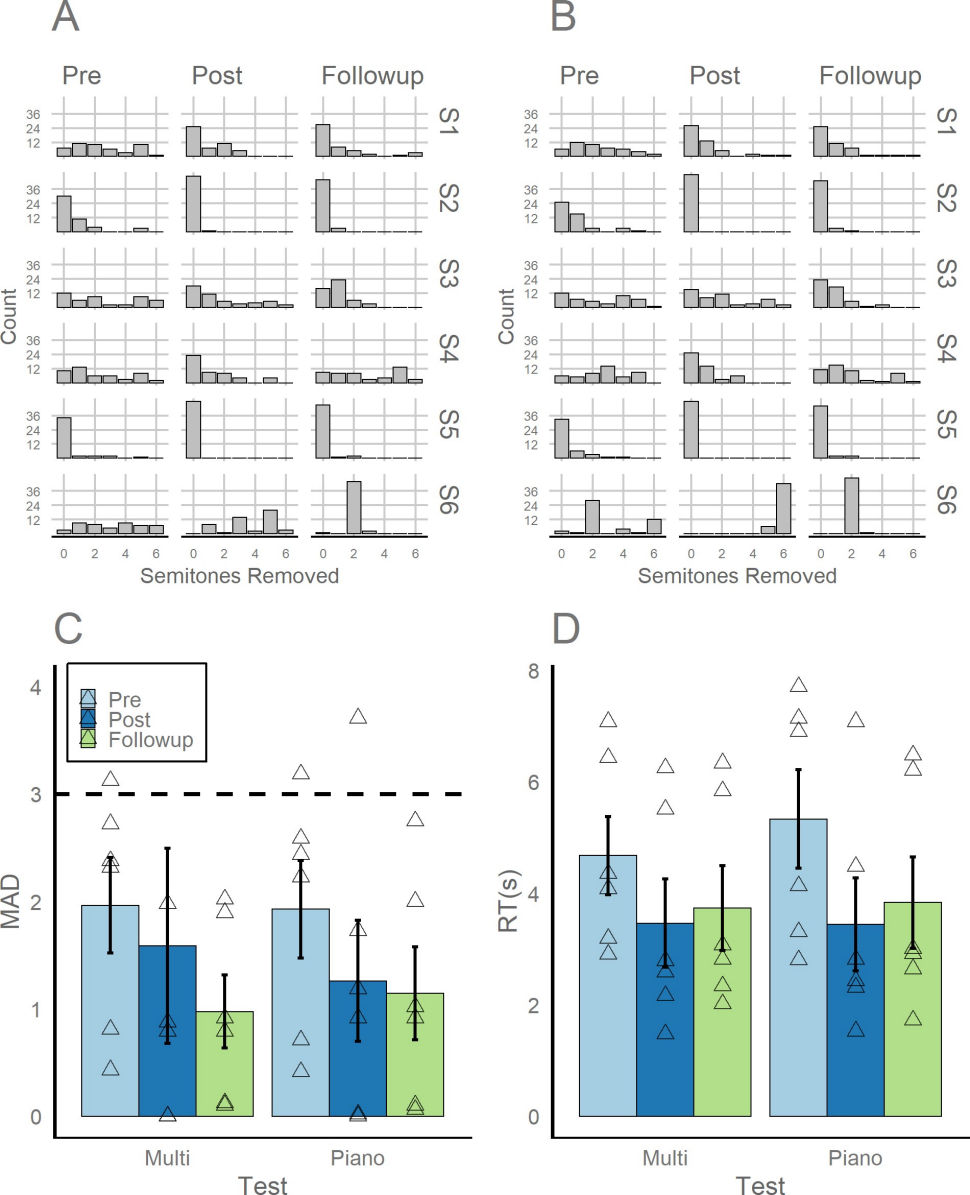
Distribution of semitone errors for each participant (S1-S6) and each testing session (Pre, Post, and Follow-Up) for the Chicago Test split by the Piano Block (A) and Multiple Timbre Block (B). Mean absolute deviation across all participants for this test is represented in panel C, whereas mean response time across all participants for this test is represented in panel D. The dashed line in Panel C represents a MAD value that would be expected by random guessing (i.e., uniform distribution of errors ranging from 0 to 6 semitones removed from the correct note).

Despite not granting credit for semitone errors on the Chicago Test, we observed higher performance compared to the UCSF Test, likely because the Chicago Test was self-paced. Participants achieved 33.3% accuracy for the Piano Block (MAD: 1.96, *SE*: 0.45) and 29.2% accuracy for the Multiple Timbre Block (MAD: 1.98, *SE*: 0.44). Participants’ mean response times (RTs) were 5.33s (*SE*: 0.88s) for the Piano Block and 4.67s (*SE*: 0.70s) for the Multiple Timbre Block, which was slower than the fixed presentation rate of notes in the UCSF Test. The distribution of responses for each participant are represented Fig 2A (Piano Block) and Fig 2B (Multiple Timbre Block), with mean accuracy represented in Fig 2C and mean response time represented in Fig 2D. All participants’ accuracy and RTs are additionally reported in Table 2.

### 3.2 Training

The complete training data are available on Open Science Framework. Here, we focus on performance from the end-of-week tests (WT for First and Second Phases, NTKT for Second Phase). The described trends in performance over the course of training are qualitative. Because of the small sample size, inferential statistics were not run on group means over time.

#### 3.2.1 Isolated note classification

**Fig 3:**
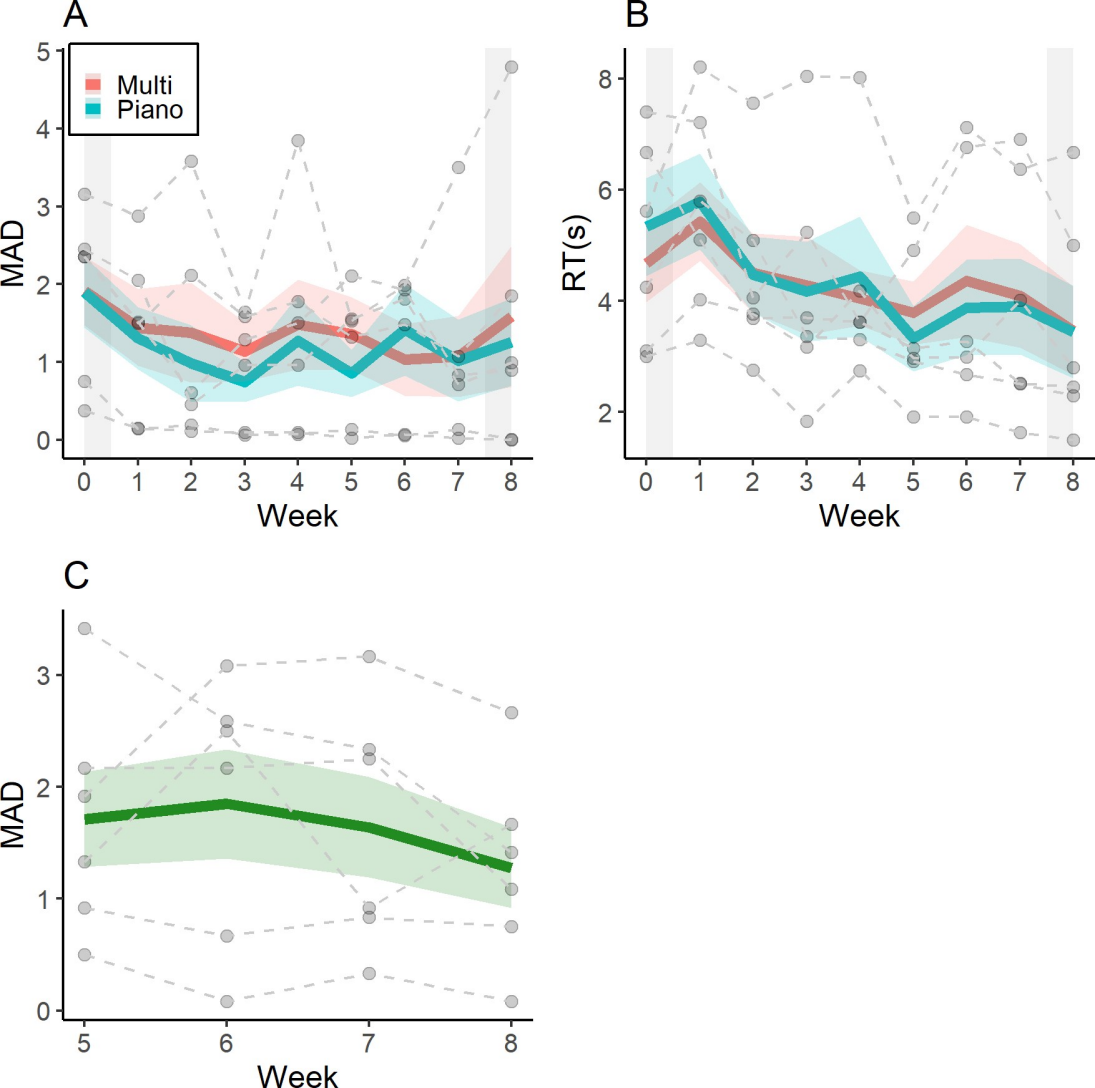
Averaged results from the weekly assessments during training. The weekly test (WT) assessment is separated by mean absolute deviation (A) and response time (B). For Panels A and B, the shaded area to the left (i.e., Week 0) represents pretest performance, whereas the shaded area to the right (i.e., Week 8) represents immediate posttest performance. The name that key test (NTKT), which is represented in Panel C, is represented in terms of mean absolute deviation. Ribbons around the mean lines represent ± 1 standard error of the mean. Individual participant trajectories are represented by the dashed grey lines.

Participants displayed a qualitative improvement in classifying isolated notes without feedback over the course of training. After the first week of training, participants correctly categorized 46.2% of notes for the Piano Block (*MAD*: 1.33, *SE*: 0.40) and 49.0% of notes for the Multiple Timbres Block (*MAD*: 1.43, *SE*: 0.50), which already represented an improvement from pre-training performance. By the end of the seventh week of training, performance had increased to 55.2% of notes for the Piano Block (*MAD*: 1.02, *SE*: 0.52) and 55.6% of notes for the Multiple Timbres Block (*MAD*: 1.07, *SE*: 0.52). Four of the six participants displayed improvements in accuracy from Week 1 to Week 7, whereas five of the six participants displayed lower MAD in the same timeframe. In terms of response time, participants were nominally *slower* in classifying notes compared to the pre-training assessment after the first week of training. Participants took 5.78s (*SE*: 0.87s) to respond to notes in the Piano Block and took 5.42s (*SE*: 0.71s) to respond to notes in the Multiple Timbres Block. By the end of the seventh week, however, participants were responding more quickly on average, taking 3.90s (*SE*: 0.86s) to respond to notes in the Piano Block and 4.09s (*SE*: 0.93s) to respond to notes in the Multiple Timbres Block. All participants exhibited faster response times from Week 1 to Week 7. Fig 3A plots MAD as a function of training week, while Fig 3B plots response time as a function of training week.

#### 3.2.2 Key signature classification

Despite only being administered four times (with each administration only consisting of 12 trials), we observed qualitative improvements in key signature classification over the course of training. In the first administration of the NTKT, participants correctly identified the key signature on 34.7% of trials (*MAD*: 1.71, *SE*: 0.42). By the final administration of the NTKT, participants correctly identified the key signature on 41.7% of trials (*MAD*: 1.28, *SE*: 0.36). Four of the six participants displayed increased accuracy and smaller MAD from the first administration on Week 5 to the final administration on Week 8. Fig 3C plots MAD for the NTKT as a function of week.

### 3.3 Post-training

#### 3.3.1 Immediate test

Within one week after training had ended, we retested all participants inside the lab on the UCSF Test and an AP test not administered prior to training (the UCSD Test) [23]. Participant S2 showed considerable improvement on the UCSF Piano and Sine Tests, scoring 34.5 on the Piano Test (MAD: 0.05) and 29.5 on the Sine Test (MAD: 0.20)–a level that was above the cutoff for *AP-1* ability. Participant S5 also showed substantial improvements, scoring 24.75 on the Piano Test (MAD: 1.32) and 17.75 on the Sine Test (MAD: 1.89), though participant S5 missed the *AP-1* qualification by 6.75 points and missed the *AP-4* qualification by 2.75 points. All other participants missed the cutoff for *AP-1* by at least 13 points and the cutoff for *AP-4* by at least 10.75 points (Table 2). On the UCSD Test, Participant S2 scored 94.44% (MAD: 0.03), while Participant S5 scored 97.22% (MAD: 0). The discrepancy between accuracy and MAD for participant S5 was because one note was not labeled at all. As such, it was counted as incorrect but could not be used in the calculation of MAD. This level of performance on the UCSD Test qualifies both participants as AP possessors based on previous interpretations of this test (using an 85% accuracy cutoff for conservative AP inclusion) [23]. As a comparison, the third highest scoring participant (S1) achieved 44.4% accuracy (MAD: 1.24), which was above chance by over 36 percentage points (represented by 1/12, or 8.33%) but below typical thresholds used to identify AP. The distribution of responses for each participant on the UCSD Test are represented Fig 1C, with average MAD represented in Fig 1D.

This pattern of results extended to the Chicago Test as well, with participants S2 and S5 displaying virtually perfect performance on both the Piano and Multiple Timbre Blocks. Participant S2 achieved 97.9% accuracy on the Piano Block (MAD: 0.02) and 100% on the Multiple Timbre Block (MAD: 0), while Participant S5 achieved 100% on both the Piano Block and Multiple Timbre Block (MAD: 0). For reference, the third highest performing participant (S1) scored 52.1% on the Piano Block (MAD: 0.92) and 54.2% on the Multiple Timbre Block (MAD: 0.88), which demonstrates above-chance performance but does not reach typical genuine AP thresholds.

Given that the present experiment was uses a case study [18] or “small *n*” [19] design, treating the individual as the unit of analysis, inferential statistics on mean improvements from Pretest to Immediate Posttest are not reported. However, we did assess improvement at the level of the individual for each test (Chicago Test and UCSF Test). This involves calculating the number of correct and incorrect trials for each test at each time point, which was then analyzed using a Bayesian equivalent of a 2 x 2 contingency table in JASP [30]. The resulting Bayes Factor (BF10) represents the relative evidence in favor of the alternative hypothesis (change in distribution of correct and incorrect responses) over the null hypothesis. For example, a BF10 of 12 would mean that the data are 12 times more likely to be observed under the alternative hypothesis compared to the null hypothesis.

The results from the analyses are reported in Table 3 (top). For the Piano Block of the Chicago Test, five of the six participants displayed moderate evidence (which we define as a BF10 > 5) of improvement from pretest to the immediate posttest. Four of the six participants displayed evidence of improvement in the Multiple Timbres block. In contrast to the general success of participants in the Chicago Test, only one participant (S2) demonstrated evidence of improvement on the Piano and Sine components of the UCSF Test.

**Table 3:** Analyses of improvements from pretest to posttest (top) and follow-up (bottom) for each participant for the Chicago Piano (C-P) and Multiple Timbre (C-MT) Blocks, as well as the UCSF Piano (UCSF-P) and Sine (UCSF-S) Tests.

*Pretest—Posttest* Participant	C-P	C-MT	UCSF-P	UCSF-S
S1	9.732	17.08	0.413	0.317
S2	6465	1.12e7	7.27e6	9.90e4
S3	5.643	2.356	0.150	0.664
S4	11.49	1555	0.834	0.170
S5	2004	4834	1.325	1.125
S6	0.334	0.163	0.938	0.226
*Pretest—Follow-up* Participant	C-P	C-MT	UCSF-P	UCSF-S
S1	31.17	9.732	0.226	0.095
S2	75.76	999.5	10.35	1772
S3	2.088	327.0	0.125	0.193
S4	0.207	0.454	0.186	0.317
S5	15.09	10.66	9.619	1.741
S6	0.162	0.163	0.203	0.467

Note: The analyses reflect Bayesian equivalents of 2x2 contingency tables. The values (BF10) represent the relative evidence for the alternative hypothesis compared to the null hypothesis. For each participant, correct and incorrect trials are tallied for each test. The change in the distribution of correct and incorrect trials from pretest to immediate posttest (top) and from pretest to the follow-up test (bottom) are used to calculate a test statistic.

#### 3.3.2 Follow-up test

To assess the stability of AP category learning, we retested all participants approximately four months (*M* = 128.17 days, *SD* = 6.71 days, range of 117–134 days) after training had ended. No participant had reported actively rehearsing pitch-label associations in the time between the immediate AP posttests and the follow-up tests. Previous AP training research has been criticized for not following up with participants after training has ended to see how category learning is retained [14], as genuine AP possessors appear to have relatively stable categories that do not require explicit maintenance (though see [17,31,32] for alternate views). We administered the same AP assessments given to participants in the posttest (UCSF Test, the UCSD Test, and the Chicago Test).

Results from the follow-up AP tests supported the conclusion that Participants S2 and S5 retained stable performance across all AP assessments. For the UCSF Test, Participant S2 still passed the *AP-1* cutoff, scoring 32.75 on the Sine Test and 30.75 on the Piano Test. Participant S5 retained high performance (13.75 on Sine, 24.75 on Piano), but was still below the *AP-1* cutoff by 10.5 points and missed the *AP-4* designation by 2.75 points. For the UCSD Test, Participant S2 scored 77.78% (MAD: 0.22) and Participant S5 scored 91.67% (MAD: 0.31). Even though Participant S2 fell below the 85% cutoff used as a conservative inclusion measure in prior studies, S2 never missed by more than one semitone. Adopting the more liberal inclusion measures from prior studies that have used the UCSD Test (in which semitone errors are allowed), Participant S2 would still be categorized as possessing AP [23]. It should be noted that Participant S2 also took the UCSD Test again approximately 16 months post-training for a separate study and achieved 88.89% accuracy (MAD: 0.14) not including semitone errors as correct. Finally, for the Chicago Test, Participants S2 and S5 scored identically in terms of percentage correct (93.75% on the Piano Block, MADs of 0.06 and 0.10, respectively) and 91.67% on the Multiple Timbre Block, MADs of 0.11 and 0.13, respectively).

The third highest performing participant was once again Participant S1, who displayed clear above chance performance on the UCSD and Chicago Tests but did not reach typical thresholds for defining AP. Participant S1 scored 63.9% on the UCSD Test (75% when including semitone errors as correct), 56.3% (MAD: 1.02) on the Piano Block of the Chicago Test, and 52.1% (MAD: 0.91) on the Multiple Timbres Block of the Chicago Test.

Similar to the immediate posttest analyses, we assessed participant retention in the follow-up test by comparing pretest performance to follow-up performance for the tests that were administered at those time points (Chicago Test and UCSF Test). These analyses (Table 3, bottom), which used Bayesian equivalents of 2 x 2 contingency tables in JASP [30], were done on the individual (not group) level. For the Piano Block of the Chicago Test, only three participants (S1, S2, and S5) displayed at least moderate evidence of improved performance compared to pretest. For the Multiple Timbres block of the Chicago Test, four of the six participants (S1, S2, S3, and S5) displayed evidence of better performance relative to pretest. Results from the UCSF Test were once again more conservative. Only participant S2 displayed consistent evidence for improved performance on both the Piano and Sine Tests relative to pretest. Participant S5 displayed moderate evidence for improved performance on the Piano Test relative to pretest. No other participant demonstrated meaningful improvements.

### 3.4 Comparisons with external studies

Unlike previous training studies [7,11–13,15], AP performance in the present study was assessed with three separate tests from three separate research groups. Two of these tests–the UCSF Test and the UCSD Test–have been externally administered in a number of previous studies [22,33–36], which makes their interpretation (in terms of AP thresholds) more straightforward. Yet, we acknowledge that these two tests may be limited in assessing the full dimensions of AP by adopting a fixed note presentation rate. Individuals who can keep up with the presentation rate will cluster together as an “AP group” (regardless of individual variation in speed) and individuals who are not able to keep up with the presentation rate will cluster together as a “non-AP group” (regardless of individual variation in speed). As such, individuals who may exhibit some intermediary AP ability will be pulled toward one of these two groups depending on whether their note classification speed is sufficient to keep up with the particular test, possibly exaggerating the appearance of a dichotomous nature to AP (those that have and those that do not have AP).

In this sense, the untimed Chicago Test provides a richer means of capturing variations in AP performance (including at the highest levels of AP performance)—without imposing strict time limits, it is possible to measure the accuracy of classification as well as the time it takes to classify notes, making it jointly possible to evaluate both speed and accuracy. To directly test whether a simultaneous consideration of speed and accuracy supports or challenges the observation that Participants S2 and S5 were behaviorally indistinguishable from genuine AP possessors post-training, we directly compare the data from the present experiment with an influential prior investigation of AP (*n* = 51), hereafter referred to as the “McGill Test” [37]. The McGill Test is particularly well-suited for comparisons with the Chicago Test because (1) both were administered on the computer, (2) both required participants to click on one of twelve provided note categories, arranged in a circular fashion, (3) both did not adopt a “timeout” window or present notes at a fixed rate, and (4) both have associated data that span a full range of performance profiles (spanning from perfect to chance performance). These parallels make it possible to better interpret the results from the present experiment in a broader context treating AP as a more distributed ability, as well as better assess whether our highest-performing participants were comparable to the highest-performing AP participants in this prior investigation.

To facilitate the comparison between the Chicago Test and the McGill Test, we created an index that incorporated both MAD and log response time (logRT), such that slower RTs would be penalized relative to faster RTs. This index, which was specifically created by adding 10 to an individual’s MAD and then multiplying this number by their logRT, was previously shown by the authors of the McGill Test to be sensitive to gradations in AP ability, capturing the nominal categories of “AP” (near perfect and fast), “non-AP” (near random and slow), and intermediate AP ability (characterized by above chance but far from perfect performance) [37].

To compare the performance of participants in the present experiment to participants in the previous study, we adopted the following procedure. First, we extracted the McGill Test data by digitizing the scatterplot (represented as Figure 7 in their paper). This provided us with all 51 participants’ index values as well as their overall accuracy (0–100%). Second, we ran a *k*-means clustering algorithm in R on just the McGill Test data (i.e., excluding the present participants) to define three groups (nominally: *genuine-AP*, *pseudo-AP*, and *non-AP*). Third, we used a naïve Bayes classifier in R, calculated with the package “e1071” to predict the posterior probabilities of each of our six participants belonging to the genuine, pseudo, and non-AP groups for each time point (pre-training, immediate posttest, and follow-up). One benefit of the naïve Bayes classifier is the posterior probability provides a more graded view of group membership (e.g., an individual may be nominally placed in one group yet have a more distributed posterior probability of belonging to each group).

**Fig 4:**
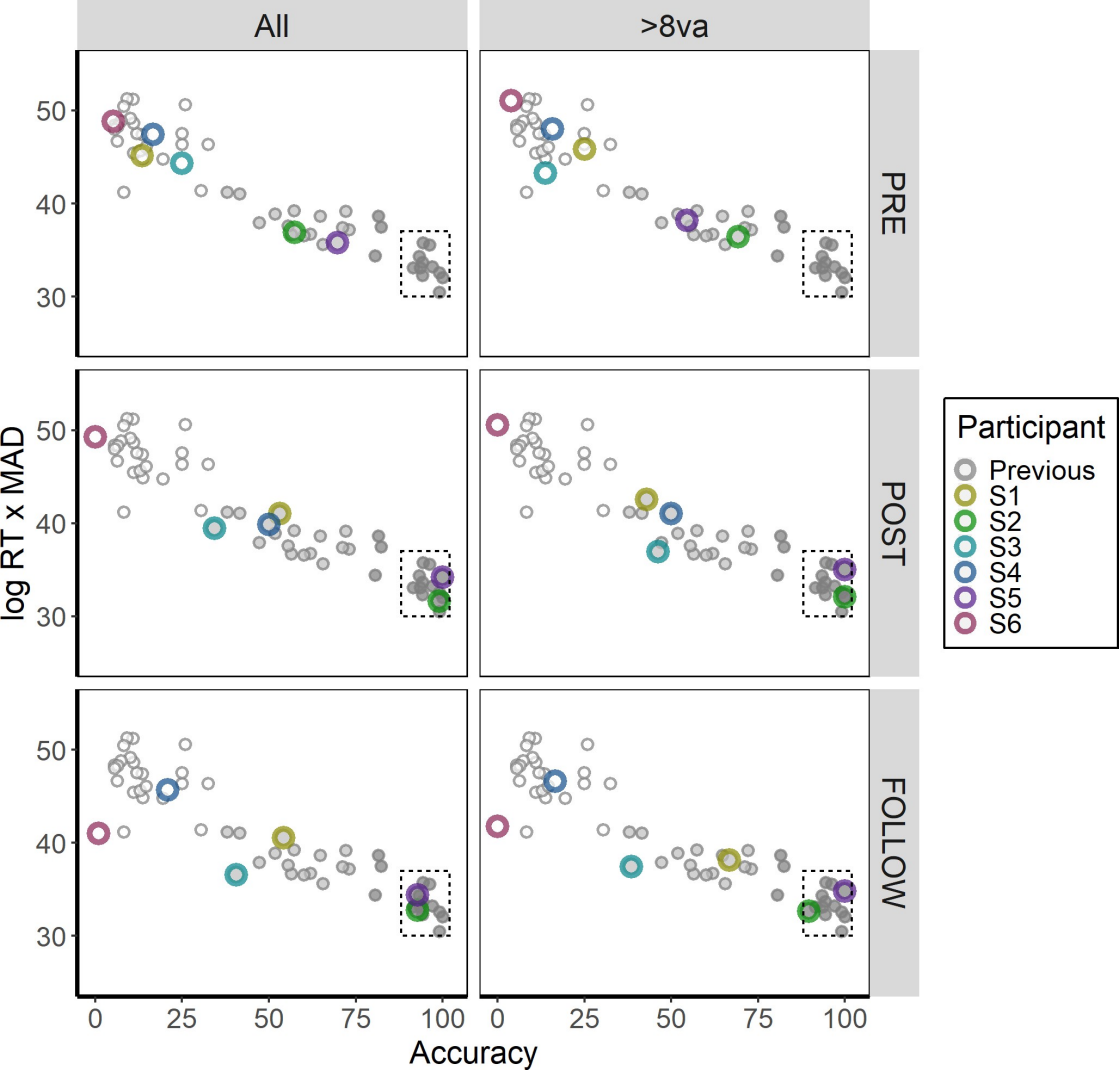
Comparison of the Chicago Test (combined performance on the Piano and Multiple Timbres Blocks) of the present experiment with Bermudez and Zatorre (2009), separated by testing session (Pre, Post, and Follow-Up). The left column (“All”) represents performance across all trials, while the right column (“>8va”) represents performance on trials in which there was more than an octave separating the heard note from the previous note. An index consisting of mean absolute deviation and log response time is represented on the y-axis, while percent correct is represented on the x-axis. Prior to training, no participant was classified in the highest (“genuine”) AP group for either analysis. In the immediate test post-training, as well as in the follow-up test four months after training, participants S2 and S5 performed indistinguishably from the highest AP performers from Bermudez and Zatorre (2009), bounded by the dashed box (lower right hand corner) of each panel.

Fig 4 (left column) plots the index value (logRT and MAD) against note classification accuracy, overlaying the six participants from the present experiment onto the extracted McGill Test data. Prior to training (“PRE”), Participant S2 and S5 were clearly distinguishable from the other four participants in terms of both the index value and mean accuracy. Yet, the naïve Bayes classifier placed both participants into the intermediate (“pseudo-AP”) group (posterior probability of 1 for S2, posterior probability of 0.990 for S5). All other participants were classified in the lowest (“non-AP”) group (posterior probability of 1).

In the immediate posttest (“POST”), Participants S2 and S5 were both classified as belonging to the highest (“genuine”) AP Group (posterior probability of 1). Participants S1, S3, and S4 were all classified as belonging to the pseudo-AP group (posterior probabilities of 1, 0.987, and 1, respectively), and Participant S6 was classified in the non-AP group (posterior probability of 1). These results were generally consistent in the follow-up test (“FOLLOW”), apart from Participant S4 whose performance sufficiently worsened to be classified in the non-AP group (posterior probability of 1). Participants S2 and S5 were still classified as belonging to the genuine-AP group, Participants S1 and S3 were still classified as belonging to the pseudo-AP group, and Participant S6 was still classified as belonging to the non-AP group (all posterior probabilities of 1). Overall, these analyses suggest that Participants S2 and S5 were performing sufficiently well post-training to be classified within the highest AP Group, *even though their performance prior to training was distinguishably lower than the highest*, *genuine-AP group*.

We acknowledge that defining three clusters may be viewed as arbitrary. However, it should be noted that doubling the number of clusters (*k* = 6) does not change the interpretation of the highest-performing participants (S2 and S5), despite making the threshold for the highest AP group more conservative. Additionally, using a visual inspection approach to define the highest cluster of AP performers, represented by the dotted boxes in Fig 4, also does not change the interpretation of Participants S2 and S5.

### 3.5 Restricted analysis

One notable difference between the Chicago Test and other external tests of AP -including the UCSD and McGill Tests—is that the Chicago Test randomly selects a note on each trial regardless of its octave. In contrast, the UCSD and McGill Tests *interleave* octaves on successive trials, ensuring that there is at least one octave separating two adjacent notes. While this difference in design may appear trivial, the rationale for using an interleaved octave approach is to disrupt relative pitch cues (e.g., see Bermudez & Zatorre, 2009). While there are valid counterarguments to this reasoning (as interleaving octaves does not inherently remove the possibility for using relative pitch cues, and rapid octave changes have been shown to disrupt performance even among “genuine” AP possessors [38]), to promote consistency with these external studies we carried out a more limited analysis in which we only considered trials that were separated from the previous trial by more than one octave.

The results are plotted in Fig 4 (right column). Prior to training, Participants S2 and S5 could still be differentiated from the other participants; however, they were classified in the pseudo-AP group (posterior probability of 0.997 for S2, posterior probability of 1 for S5). In the immediate posttest, Participants S2 and S5 were classified in the genuine-AP group (posterior probability of 1). Participants S1, S3, and S4 were all classified in the pseudo-AP group, though the rankings of these participants differed from the full trial analysis. Specifically, Participant S1 –who was the most accurate pseudo-AP participant when considering all trials–was the *least* accurate pseudo-AP participant in this analysis (posterior probability of 0.947 for pseudo-AP and 0.053 for non-AP). This suggests that Participant S1 may have been using a strategy in the immediate posttest that was particularly harmed by octave changes. In the follow-up test, however, Participant S1 displayed improvements in accuracy (42.9% to 66.7%), MAD (1.21 to 0.63), and response time (7.19s to 5.08s) and was thus the highest-performing pseudo-AP participant (100% posterior probability of pseudo-AP), suggesting a possible shift in strategy or improved generalization across octaves. Participants S2 and S5 were still classified in the genuine-AP group, Participant S3 was classified in the pseudo-AP group (99.9% posterior probability), and both Participants S4 and S6 were classified in the non-AP group (all posterior probabilities of 1).

Taken together, these comparisons with the McGill Test data highlight the importance of jointly using response time and accuracy to gain a more comprehensive understanding of variability in AP performance. In particular, the second analysis (examining trials that were at more than one octave removed from the previous trial) suggests that Participant S1 particularly was harmed when notes changed by more than one octave, though there are several ways of interpreting these results. Regardless of the interpretation of Participant S1, the restricted analysis reported in the present section demonstrates that Participants S2 and S5 displayed performance profiles consistent with genuine AP possessors.

## Discussion

The present results challenge two theoretical assumptions regarding AP–(1) that it is a dichotomous, “all or none” ability [21], and (2) that genuine AP patterns of performance cannot emerge in post-critical-period adults [1]. We interpret the present experiment as providing strong evidence for adult AP acquisition; however, we acknowledge that this is not the only interpretative framework that may explain these data. Below, we outline two “non-learning” alternative explanations that could be proposed and discuss why they are insufficient in explaining our results.

The first non-learning explanation is that our successful participants were always “AP possessors,” and therefore the training program merely revealed an inherent, latent ability rather than reflected genuine learning. This kind of explanation comes from a broader framework treating AP as an innate perceptual ability, requiring essentially no environmental input and therefore not being restricted to a critical period of development [39–41]. At first glance, this possibility seems to be partially supported by the pretest results, as Participants S2 and S5 were already distinguishable from the other participants in terms of AP performance *prior to training*. However, the notion that both were already “AP possessors” (as typically defined) seems unlikely for two reasons. First, the data-driven approach to defining the highest cluster of AP performance (“genuine AP”) demonstrated that, prior to training, Participants S2 and S5 were not sufficiently fast and accurate to be considered a part of this group (0 probability for Participant S2, 0.01 probability for Participant S5). Second, both participants had extensive musical backgrounds, and thus had extensive opportunities to learn note-label associations. Specifically, Participant S2 and S5 began musical instruction at 7 and 6 years old, respectively, and played their primary instrument for 8 and 20 years, respectively. If AP reflects an innate ability that only nominally requires environmental shaping (i.e. to learn the conventional names of Western musical notes), then both participants should have performed with sufficient speed and accuracy to be labeled as AP possessors prior to training, given their considerable prior experience associating pitches with their respective note names. In other words, prior to the present training regime, these participants had early musical training and experienced extensive pitch-label associations during regular musical exercises and practice but still did not have AP as adults, which would require the formation of a new theory of AP manifestation rather than AP acquisition.

The second non-learning explanation is that our successful participants never possessed AP (even after training), but rather found some means of simulating AP level performance through strategies that did not involve the actual perceptual classification of the twelve note categories. In particular, participants may have been able to memorize one or two absolute pitches and then used relative pitch to categorize the remaining pitches–a phenomenon that has been discussed as distinct from genuine AP for the better part of a century [42]. Yet, given the history of this distinction in the literature, many AP assessments have adopted methodologies to minimize relative pitch strategies, primarily related to note classification speed and the intervallic distance between consecutive notes. Yet, these factors did not appear to hinder our highest-scoring participants’ performance post-training. Even when using a more nuanced approach (simultaneously weighing accuracy and response speed for the untimed Chicago Test), which is more sensitive to graded AP performance profiles, we found strong evidence that Participants S2 and S5 were indistinguishable from a prior group of “genuine” AP possessors post-training, even when limiting the analyses to notes that were separated by more than one octave. Thus, if one wants to claim that what we observed is not genuine AP, then either the current definition of AP or the ways in which AP is tested need to be fundamentally reconsidered.

Yet, the present results must also be interpreted in more pragmatic terms. The fact that only two of six musically trained participants with exceptional auditory memory abilities performed with sufficient speed and accuracy post-training to be indistinguishable from “genuine” AP possessors may lead one to conclude that adult AP acquisition is so difficult or dependent on sufficiently rare individual characteristics that it is of little theoretical import. This kind of argument, however, also depends on assumptions that other aspects of the experiment, such as the nature of the training paradigm, were optimized to produce AP learning. Yet, the training paradigm could not have been optimized simply because such training is not yet understood sufficiently well. For example, the first phase of training was designed to ease participants into learning–that is, to not present too formidable a recognition task. However, this decision may have *hindered* AP learning because the tasks could largely be completed using relative pitch cues, which might have led some participants to use strategies that would not serve well for actual AP performance when such cues were not available. In particular, the speeded tasks in the first phase only used “white key” notes and thus could have encouraged participants to hear the target notes in a relative tonal context of C major. The “Accuracy Training” task, which was a staple of training for all eight weeks, may not have been successful in erasing an auditory trace between trials, as we only played 1000 ms of noise between trials. This means that some participants could have used relative pitch from the feedback of previous trials to improve performance, resulting in a continued reliance on relative pitch strategies even during the AP Tests when such strategies would be rendered ineffective. While these concerns do not apply to the AP Tests we administered to gauge AP learning (as no feedback was provided at any point during these tests), it is possible that a training paradigm that discouraged the use of relative pitch strategies from the outset may have resulted in better AP learning than what was observed for some of the participants in the present experiment, even if it resulted in initially worse performance during training.

Overall, the present results support a *skill acquisition* theory of AP, in that some individuals can improve their AP abilities following explicit perceptual training to the point where their performance is indistinguishable from AP possessors whose abilities manifested early in life. Yet, it is misleading to think that a *skill acquisition* theory of AP cannot be partly reconciled with the more dominant theories of AP acquisition. In the following paragraphs, we highlight how the results from the present experiment may be integrated with both the *critical period* and *innate* theories of AP acquisition, particularly when conceptualizing AP as a continuous and non-dichotomous ability.

Under the *critical period* theory, AP acquisition is almost exclusively confined to an early window of development. The “cutoff” for being able to acquire AP is not likely a strict age, but rather reflected as a decreasing probability of acquisition as a function of aging [5], although the causal mechanism of aging is not specified clearly. Thus, finding two successful adult AP learners is not technically incompatible with the *critical period* theory; however, observing a 33% success rate among an adult sample–even if that sample was non-randomly selected–would be virtually impossible given (1) the presumed rarity of AP and (2) the relative probability of acquiring AP as an adult suggested by a *critical period* framework. An important point to consider in the context of the present experiment, however, is that both successful participants began musical training at an age that would be more compatible with the *critical period* theory (i.e. younger than 8 years old). Given that both participants had experience associating musical pitches with their note labels relatively early in life, the *critical period* theory could be integrated with a *skill acquisition* theory. For example, early musical training may be necessary (but not sufficient) for developing the capacity to meaningfully refine AP ability, though the actual refinement of note categories may take on a more heterogeneous trajectory. On one end of this continuum would be individuals who acquire AP rapidly and without any reported effort [43]. On the other end of this continuum would be individuals who refine their note categories over the course of extended, concentrated practice (as was the case in the present experiment). Importantly, just because the experiences in acquiring AP differ across this continuum does not mean that individuals cannot converge on the same level of AP proficiency. However, we stress that a possible integration of the *skill acquisition* and *critical period* theories of AP such as the one described above would still require a reconceptualization of the presumed mechanisms that underlie critical periods in AP.

Additionally, the *skill acquisition* theory of AP does not necessarily suggest that any individual can develop sufficient speed and accuracy to be indistinguishable from a genuine AP possessor. In our own sample, the two successful AP learners were performing better *before training* than the third highest-performing participant *after training*, suggesting that these participants had some degree of AP at pretest that was refined over the course of training. To be clear, however, by all tests of AP and by most theoretic positions about AP, these individuals would never be considered to qualify as having AP. Importantly, under a continuous, non-dichotomous view of AP ability, such a finding is not necessarily surprising, though the present results are important in demonstrating that individuals with some degree of AP may stand to benefit the most from explicit training. Highlighting the difference between the two best performers and the rest of the participants, the third-highest performing participant was slower and more disrupted by shifts larger than an octave compared to the two best learners, suggesting that performance may have been the result of adopting a different strategy. As such, our results may also inform the *innate* theory of AP acquisition, for it is possible that only some adults can develop genuine AP levels of performance post-training. If this were the case, then it would be of great scientific interest to understand (1) the base rate of trainable adults, (2) the perceptual, cognitive, and possibly underlying genetic factors that differentiate these individuals from non-trainable adults, and (3) whether these individuals always report early musical training. Importantly, these findings suggest that it may be more appropriate to consider more general mechanisms (e.g., working memory, attention) as the “innate” component of AP rather than a specialized mechanism specifically supporting AP.

If AP is framed as a clearly defined, dichotomous ability [34], it is difficult to entertain the idea that AP can be acquired as a function of training. This is because there is no middle ground–performance should be either close to random or close to perfect. As such, the transition from “non-AP” to “AP” would represent a transition from the complete absence of an ability to the total manifestation of an ability, as if AP reflected a sudden insight into how to interpret and label auditory pitch. In contrast, when framing AP as a distributed, multidimensional ability [1,37,44], learning theories become more theoretically plausible. This is because “successful” AP learning does not need to represent a binary switch. Rather, in some cases, a rather modest degree of learning may be sufficient to result in “genuine AP” levels of performance. Yet, approaching AP as a learnable skill into adulthood has been largely dismissed in the literature, despite a growing body of research suggesting that, even among AP possessors, absolute pitch ability can be significantly strengthened or even weakened by environmental experiences (e.g., recent musical training) outside of a critical period [31,45,46], and despite the existence of some adult AP training studies that have similarly found impressive levels of note naming performance after training [12,47].

Why, then, is there a hesitancy to consider AP in a *skill acquisition* framework? One reason is that there still may be an implicit assumption that AP should still be dichotomized, even if performance lies along a continuum. In other words, there is still a tendency to treat individuals who fall on the highest end of an AP continuum as engaging in a fundamentally different process from other individuals (e.g., differentiating “genuine” from “pseudo” AP as a character trait). While this approach may be justified in some cases (e.g., to distinguish alternative strategies in note identification), it also may unnecessarily simplify AP and downplay the role of learning and plasticity in AP more generally. For example, many “non-AP” listeners are able to tell when a familiar, pitched stimulus (e.g., dial tone from telephone, censor “bleep” used in media) deviates from its typical absolute pitch, even in situations where the deviation is less than one semitone [48,49]. More recently, “non-AP” listeners were shown to possess an absolute sense of Western intonation (i.e., where the A above middle C is tuned to 440Hz), which was previously thought to be an ability solely afforded by possessing AP [50]. These pitch memory abilities, which can exist independently of explicit note labels, thus appear to offer a potentially important insight into understanding the formation and maintenance of absolute pitch abilities in humans.

A second reason why there may be hesitancy in accepting a *skill acquisition* framework is the lack of conclusive previous empirical evidence that AP can be trained in adults [43]. Of course, inferring that AP cannot be trained in adults based on the absence of prior evidence represents an acceptance of a null hypothesis, and as such, it is impossible to know whether failures of previous training studies were due to participant selection, the length or nature of the training regime, the operationalization of AP ability, or other factors.

Indeed, the positive results obtained in the present experiment point may be partly attributed to the selection of participants based on exceptional auditory memory abilities. Recent research has associated aspects of auditory WM and STM with pitch memory performance in a wide variety of settings, such as (1) absolute memory for familiar musical recordings [24], (2) the rapid and explicit training of AP among previously naïve adults [15], and (3) MAD among a “genuine AP” population for perceptually challenging notes [51]. Moreover, “genuine AP” possessors appear to have an enhanced auditory (but not visual) digit span relative to musically matched controls [29], which further suggests that general auditory memory abilities may be a particularly important factor in understanding absolute pitch representations, including the explicit training of AP. As such, future investigations into adult AP learning may benefit from dissociating auditory WM/STM abilities from (early) musical training, as this could provide important insights into both the *critical period* and *skill acquisition* theories of AP.

The present results give empirical weight to the theoretical treatment of AP as an auditory skill rather than a static talent. This viewpoint is supported through providing the most conclusive demonstration to date that AP ability can be improved through training to the point of being indistinguishable from “genuine AP” within some adults. It is also important to consider that these levels of AP performance were achieved with eight weeks (approximately 32 hours) of adult training, which is far less than the amount of training that is thought to be required for the explicit learning of AP in childhood based on prior research [52].

While the present study is limited in sample size, the demonstration that even two adults can, with moderate training, reach genuine AP levels of performance is theoretically important. No prior published study has demonstrated a comparable level of successful adult AP learning and long-term retention. In a single-session adult AP training study, learning was found to be stable for at least six months following training, though these participants did not reach typical AP thresholds and retention was only tested on a subset of participants [15]. As such, we stress the importance of these findings as proof-of-concept that adult AP acquisition is possible and that learning remains stable months after explicit training has ended. To this end, *any* demonstration of successful AP learning by an adult will inform the discussion of the underlying mechanisms of AP acquisition and maintenance. However, given the limited sample size, we emphasize that no claims can be supported related to the relative proportion of adults who may display AP-like performance post training.

While the present results cannot refute either the *critical period* or *innate* theories of AP acquisition, they suggest that aspects of both theories should be more fully integrated with a *skill acquisition* theory of AP. This integration becomes clearer when conceptualizing AP as a distributed and multifaceted ability rather than a static and dichotomous ability. Overall, it is our hope that these results will refocus future inquiry regarding AP to treat the ability as distributed and at least partly plastic, even into adulthood, as this refocusing will lead to a more complete understanding of how humans perceive and remember absolute pitch information more generally.

## References

[1] Takeuchi AH, Hulse SH. Absolute pitch. Psychol Bull. 1993;113: 345–361. 10.1037/0033-2909.113.2.345 8451339

[2] Deutsch D. Absolute Pitch In: Deutsch D, editor. The Psychology of Music. 3rd ed San Diego, CA: Academic Press; 2013 pp. 141–182. 10.1016/B978-0-12-381460-9.00005-5

[3] Ward WD, Burns EM. Absolute pitch In: Deutsch D, editor. The Psychology of Music. 1st ed San Diego, CA: Academic Press; 1982 pp. 431–451.

[4] Crozier JB. Absolute pitch: Practice makes perfect, the earlier the better. Psychol Music. 1997;25: 110–119. doi:0803973233

[5] Levitin DJ, Zatorre RJ. On the Nature of Early Music Training and Absolute Pitch: A Reply to Brown, Sachs, Cammuso, and Folstein. Music Percept. 2003;21: 105–110. 10.1525/mp.2003.21.1.105

[6] Hartman EB. The influence of practice and pitch-distance between tones on the absolute identification of pitch. Am J Psychol. 1954;67: 1–14. 10.2307/1418067 13138765

[7] Cuddy LL. Practice Effects in the Absolute Judgment of Pitch. J Acoust Soc Am. 1968;43: 1069–1076. 10.1121/1.1910941 5648097

[8] Lundin RW. Can perfect pitch be learned? Music Educ J. 1963;49: 49–51. 10.2307/3389949

[9] Vianello M a, Evans SH. Note on pitch discrimination learning. Percept Mot Skills. 1968;26: 576 10.2466/pms.1968.26.2.576 5654888

[10] Russo FA, Windell DL, Cuddy LL. Learning the “special note”: Evidence for a critical period for absolute pitch acquisition. Music Percept An Interdiscip J. 2003;21: 119–127.

[11] Gervain J, Vines BW, Chen LM, Seo RJ, Hensch TK, Werker JF, et al Valproate reopens critical-period learning of absolute pitch. Front Syst Neurosci. 2013;7: 1–11. 10.3389/fnsys.2013.00001 24348349 PMC3848041

[12] Brady PT. Fixed‐Scale Mechanism of Absolute Pitch. J Acoust Soc Am. 1970;48: 883–887. 10.1121/1.1912227 5480385

[13] Heller MA, Auerbach C. Practice effects in the absolute judgment of frequency. Psychon Sci. 1972;26: 222–224.

[14] Rush M. An experimental invesitagtion of the effectiveness of training on absolute pitch in musicians. 1989. p. 401.

[15] Van Hedger SC, Heald SLM, Koch R, Nusbaum HC. Auditory working memory predicts individual differences in absolute pitch learning. Cognition. 2015;140: 95–110. 10.1016/j.cognition.2015.03.012 25909580

[16] Heald SLM, Van Hedger SC, Nusbaum HC. Understanding Sound: Auditory Skill Acquisition. Psychology of Learning and Motivation—Advances in Research and Theory. 2017 10.1016/bs.plm.2017.03.003

[17] Hedger SC, Heald SLM, Nusbaum HC. Absolute Pitch May Not Be So Absolute. Psychol Sci. 2013;24: 1496–1502. 10.1177/0956797612473310 23757308

[18] Crowe S, Cresswell K, Robertson A, Huby G, Avery A, Sheikh A. The case study approach. BMC Med Res Methodol. 2011;11: 1–9. 10.1186/1471-2288-11-1 21707982 PMC3141799

[19] Smith PL, Little DR. Small is beautiful: In defense of the small-N design. Psychon Bull Rev. 2018;25: 2083–2101. 10.3758/s13423-018-1451-8 29557067 PMC6267527

[20] Mathôt S, Schreij D, Theeuwes J. OpenSesame: An open-source, graphical experiment builder for the social sciences. Behav Res Methods. 2012;44: 314–324. 10.3758/s13428-011-0168-7 22083660 PMC3356517

[21] Athos EA, Levinson B, Kistler A, Zemansky J, Bostrom A, Freimer N, et al Dichotomy and perceptual distortions in absolute pitch ability. Proc Natl Acad Sci U S A. 2007;104: 14795–14800. 10.1073/pnas.0703868104 17724340 PMC1959403

[22] Baharloo S, Johnston PA, Service SK, Gitschier J, Freimer NB. Absolute Pitch: An Approach for Identification of Genetic and Nongenetic Components. Am J Hum Genet. 1998;62: 224–231. 10.1086/301704 9463312 PMC1376881

[23] Deutsch D, Henthorn T, Marvin E, Xu H. Absolute pitch among American and Chinese conservatory students: Prevalence differences, and evidence for a speech-related critical period. J Acoust Soc Am. 2006;119: 719 10.1121/1.2151799 16521731

[24] Van Hedger SC, Heald SLM, Nusbaum HC. Long-term pitch memory for music recordings is related to auditory working memory precision. Q J Exp Psychol. 2017; 1–13. 10.1080/17470218.2017.1307427 28856955

[25] Brainard DH. The Psychophysics Toolbox. Spat Vis. 1997;10: 433–436. 10.1163/156856897X00357 9176952

[26] Pelli DG. The VideoToolbox software for visual psychophysics: transforming numbers into movies. Spatial vision. 1997 pp. 437–442. 10.1163/156856897X00366 9176953

[27] Macmillan NA, Creelman CD. Detection theory: A user’s guide. 2nd ed Mahwah, New Jersey: Lawrence Erlbaum Associates; 2005.

[28] Heald SLM, Van Hedger SC, Nusbaum HC. Auditory category knowledge in experts and novices. Front Neurosci. 2014;8: 1–15. 10.3389/fnins.2014.00001 25202232 PMC4141216

[29] Deutsch D, Dooley K. Absolute pitch is associated with a large auditory digit span: A clue to its genesis. J Acoust Soc Am. 2013;133: 1859–1861. 10.1121/1.4792217 23556554

[30] Marsman M, Wagenmakers EJ. Bayesian benefits with JASP. Eur J Dev Psychol. 2017;14: 545–555. 10.1080/17405629.2016.1259614

[31] Wilson SJ, Lusher D, Martin CL, Rayner G, McLachlan N. Intersecting factors lead to absolute pitch acquisition that is maintained in a “Fixed do” Environment. Music Percept. 2012;29: 285–296.

[32] Van Hedger SC, Heald SLM, Uddin S, Nusbaum HC. A Note by Any Other Name: Intonation Context Rapidly Changes Absolute Note Judgments. J Exp Psychol Hum Percept Perform. 2018; 10.1037/xhp0000536 29708383

[33] Baharloo S, Service SK, Risch N, Gitschier J, Freimer NB. Familial aggregation of absolute pitch. Am J Hum Genet. 2000;67: 755–758. 10.1086/303057 10924408 PMC1287535

[34] Athos EA, Levinson B, Kistler A, Zemansky J, Bostrom A, Freimer N, et al Dichotomy and perceptual distortions in absolute pitch ability. Proc Natl Acad Sci. 2007;104: 14795–14800. 10.1073/pnas.0703868104 17724340 PMC1959403

[35] Deutsch D, Dooley K, Henthorn T, Head B. Absolute pitch among students in an American music conservatory: Association with tone language fluency. J Acoust Soc Am. 2009;125: 2398–2403. 10.1121/1.3081389 19354413

[36] Deutsch D, Li X, Shen J. Absolute pitch among students at the Shanghai Conservatory of Music: A large-scale direct-test study. J Acoust Soc Am. 2013;134: 3853–3859. 10.1121/1.4824450 24180794

[37] Bermudez P, Zatorre RJ. A distribution of absolute pitch ability as revealed by computerized testing. Music Percept An Interdiscip J. 2009;27: 89–101. 10.1525/MP.2009.27.2.89

[38] Van Hedger SC, Heald SLM, Nusbaum HC. The effects of acoustic variability on absolute pitch categorization: Evidence of contextual tuning. J Acoust Soc Am. 2015;138: 436–446. 10.1121/1.4922952 26233042

[39] Ross DA, Marks LE. Absolute pitch in children prior to the beginning of musical training. Ann N Y Acad Sci. 2009;1169: 199–204. 10.1111/j.1749-6632.2009.04847.x 19673781

[40] Ross DA, Olson IR, Marks LE, Gore JC. A nonmusical paradigm for identifying absolute pitch possessors. J Acoust Soc Am. 2004;116: 1793–1799. 10.1121/1.1758973 15478446

[41] Ross DA, Gore JC, Marks LE. Absolute pitch: Music and beyond. Epilepsy Behav. 2005;7: 578–601. 10.1016/j.yebeh.2005.05.019 16103017

[42] Bachem A. Various Types of Absolute Pitch. J Acoust Soc Am. 1937;9: 146–151. 10.1121/1.1915919

[43] Deutsch D. 5—Absolute Pitch [Internet]. The Psychology of Music (Third Edition). 2013 10.1016/B978-0-12-381460-9.00005-5 10.1016/B978-0-12-381460-9.00005-5

[44] Vitouch O. Absolutist models of absolute pitch are absolutely misleading. Music Percept. 2003;21: 111–117.

[45] Dohn A, Garza-Villarreal EA, Ribe LR, Wallentin M. Musical activity tunes up absolute pitch ability. Music Percept. 2014;31: 359–371. 10.1525/MP.2014.31.4.359

[46] Bahr N, Christensen CA, Bahr M. Diversity of accuracy profiles for absolute pitch recognition. Psychol Music. 2005;33: 58–93. 10.1177/0305735605048014

[47] Wong YK, Lui KFH, Yip KHM, Wong AC-N. Acquiring absolute pitch is difficult but possible. bioRxiv. 2018;355933 10.1101/355933

[48] Van Hedger SC, Heald SLM, Nusbaum HC. What the [bleep]? Enhanced absolute pitch memory for a 1000 Hz sine tone. Cognition. 2016;154 10.1016/j.cognition.2016.06.001 27289485

[49] Smith NA, Schmuckler MA. Dial A440 for absolute pitch: Absolute pitch memory by non-absolute pitch possessors. J Acoust Soc Am. 2008;123: EL77–EL84. 10.1121/1.2896106 18396925

[50] Van Hedger SC, Heald SLM, Huang A, Rutstein B, Nusbaum HC. Telling in-tune from out-of-tune: widespread evidence for implicit absolute intonation. Psychon Bull Rev. 2017;24 10.3758/s13423-016-1099-1 27383616

[51] Van Hedger SC, Nusbaum HC. Individual differences in absolute pitch performance: Contributions of working memory, musical expertise, and tonal language background. Acta Psychol (Amst). 2018;191: 251–260. 10.1016/j.actpsy.2018.10.007 30347313

[52] Miyazaki K, Ogawa Y. Learning Absolute Pitch by Children. Music Percept. 2006;24: 63–78. 10.1525/mp.2006.24.1.63

